# Differences between Transdermal Fentanyl and Buprenorphine in the Elderly Hospice Patients

**DOI:** 10.1155/2018/8610538

**Published:** 2018-10-15

**Authors:** Marin Golčić, Renata Dobrila-Dintinjana, Goran Golčić, Lidija Gović-Golčić

**Affiliations:** ^1^Department of Radiotherapy and Oncology, Clinical Hospital Center Rijeka, Croatia; ^2^General Practitioner Office, Rijeka, Croatia

## Abstract

**Introduction:**

Opioids are the most important drugs in treating pain in palliative care patients. Transdermal formulations are especially useful due to their noninvasive nature and minimal interference in daily life. However, studies have shown a controversial relationship of opioids to survival and a rise in deaths associated with the use of transdermal opioids. Although applying precise doses is paramount, we have no clear recommendations for the exact equianalgesic ratio for buprenorphine patch and no recommendation for the type of transdermal opioid to use in hospice.

**Methods:**

We analyzed the differences between the transdermal fentanyl and buprenorphine group by analyzing patient characteristics and evaluating the differences in survival in hospice patients over the age of 65, from 2013 to 2017.

**Results:**

A total of 292 patients (75.8%) used fentanyl patch and 93 (24.1%) were on buprenorphine patch. Patients had virtually the same characteristics in both groups. However, when using a 1:100 buprenorphine equianalgesic ratio, there were significant differences in initial and final doses, and it seems that a 1:80 conversion rate is more accurate for elderly hospice patients. Finally, there was no difference in survival between the two groups using transdermal opioids, with or without adjuvant analgesics.

**Discussion:**

There were no differences in survival between the group using transdermal fentanyl and the group using buprenorphine in the elderly hospice population. Although adjuvant NSAIDs could be useful in the treatment of pain in terminal cancer, they do not affect survival or reduce the opioid doses, while a 1:80 equianalgesic ratio of buprenorphine might be the most appropriate in this population.

## 1. Introduction

Opioids are the essential drugs in treating pain in palliative care patients [1]. Along with sedatives, antipsychotics, and antimuscarinic drugs, they are considered as one of the four essential drugs for dying patients [2]. However, there are controversies for opioid use in the hospices, as studies have shown that opioid use is associated with both longer and shorter survival [3, 4]. One of the possible explanations for such results might lie in a choice and formulation of the opioid used, which is rarely analyzed. Transdermal (TD) opioids are generally considered as safe and effective drugs, often preferred over oral opioids, due to their noninvasive nature, minimal interference with daily activities, and the availability in patients who are unable to swallow or have otherwise poor compliance [5, 6].

TD opioids are successfully used in the hospice setting [7], both the fentanyl and the buprenorphine TD formulation. However, the two drugs have very different pharmacology [8] and are rarely directly compared. So far, there are no recommendations of whether one formulation should have an advantage over the other in the hospice setting. Of course, quality of life and the level of pain should be the primary concerns when choosing the opioid, but it is also important to assess that these drugs do not have a negative effect on the survival in the frail hospice population, especially knowing there is a rise in deaths associated with the use of TD opioids [9]. At-risk populations include cachectic cancer patients, who have impaired absorption of the TD opioids [10], the elderly due to multiple comorbidities and medicines used [11], as well as patients with hepatic impairment [12], all of which are common traits in hospice patients.

An additional issue with the TD buprenorphine is that there are no precise data for equipotency ratio of TD buprenorphine to oral morphine, which is necessary to calculate the needed dose for opioid conversion [13]. Current research usually recommends using conversion rates from 70-75:1 for cancer patients [14, 15] to 110-115:1 for the mixed population [16]. Such differences are potentially dangerous as mismanagement of the dose could lead to either ineffective pain reduction or respiratory depression, especially in frail elderly hospice population. A more precise conversion rate for this population is needed.

Since the hospice population is a mixed population, with the predominantly elderly cancer patients, we hypothesize that a conversion rate of buprenorphine of 1:100 is adequate. We also hypothesize that there are no significant differences in survival between the fentanyl and buprenorphine patches, based on our empirical experience.

## 2. Methods

The study was a retrospective analysis of the patients enrolled in the only Croatian hospice Marija K. Kozulić, from March 2013 to March 2017. Although there is a variety of definitions of an elderly patient, we used the common definition of patients aged 65 years or older [17]. For patients to be included in the research; they required an evaluation that estimated survival time is less than three months and required to have a medical confirmation that no further active treatment is possible.

We analyzed the differences between the TD fentanyl and buprenorphine by comparing patients characteristics in both groups, analyzing the use of adjuvant analgesics and sedatives, and evaluating the differences in survival between the different patient groups. TD fentanyl patches were changed every 3 days, while TD buprenorphine was changed every 4 days, as per instructions.

The peroral opioids we used were morphine, oxycodone, fentanyl, tramadol, and methadone, while the nonsteroidal anti-inflammatory drugs (NSAIDs) used in the hospice were ibuprofen, diclofenac, ketoprofen, metamizole, and paracetamol. We used McPherson's guide to convert different opioids to Oral Morphine Equivalents (OME, mg/day) [18], while for TD buprenorphine we used both 1:100 and 1:80 conversion rates. Both NSAIDs and opioids were included in the study as they were the only conventional pain medication used in our population.

The performance score used is a Croatian patient categorization system [19], where 1 denotes a completely independent patient and 4 signifies a patient completely bedridden, in the same manner as the ECOG scale [20]. Scores 2 to 3 describe patients dependent on a moderate or high degree, depending on patient's physical activity and self-care.

This research was approved by the Ethics Committee of the Marija K. Kozulić Hospice and was undertaken adhering to the highest ethical standards.

### 2.1. Statistics

We initially performed the Kolmogorov-Smirnov test, which pointed us to use nonparametric tests. Hence, Kruskal-Wallis ANOVA was used for comparing multiple independent samples, while Mann-Whitney *U* test was used to compare two independent samples. Log-rank test and Kaplan Meier method were used to analyze survival. Descriptive analysis was performed to analyze general information. We performed statistics using Statistica software 12 (StatSoft, USA).

## 3. Results

Initially, we assessed 902 patients enrolled in the hospice over the examined period of 4 years. A total of 667 were older than 65 years and were taken in further analysis. In this group of patients, 385 were using transdermal opioids (57.7%), 88 (13.2%) were using only peroral opioids, while 194 (29.1%) did not use any opioids.

The average age of our patients was 77.57 years, with 329 females (49.3%). High school was the highest level of education for 344 (51.6%) of our patients, with the majority of patients being married (N=295, 44.2%) or widowed (N=220, 32.9%).

The majority of patients (N=562, 84.3%) had cancer as their primary diagnosis, with the most common cancer being lung cancer (N=123, 18.4%), followed by gastrointestinal cancers (N=122, 18.3%) and hepatobiliary cancers (N=74, 11.1%). If the patient did not have cancer, the most likely diagnosis was the cerebrovascular injury (N=28, 4.2%). The average performance score was 3.24 (±0.92), with a total of 347 (52.1%) patients evaluated as bedridden on admission. On average, patients spent 17 days in the hospice. A total of 559 (83.8%) of the patients died in the hospice, while the rest were discharged or transferred to other institutions.

Patients used two different formulations of TD opioids, with 292 patients (75.8%) on the fentanyl patch and 93 (24.2%) on buprenorphine patch (Table 1). There were no differences between the two groups regarding average age, gender, the percentage of cancer patients, number of patient deaths, dose elevations, dose reductions, or performance score, which indicates that the two groups were very similar in patient characteristics. Additionally, we observed no differences in the concomitant use of peroral opioids, NSAIDs, sedatives, or antipsychotics.

Although we did not assess the dose of all NSAIDs due to difficulties in assessing generalised NSAIDs dose, we did analyze the paracetamol dose between the two groups and there were no significant differences recorded as 68 fentanyl patients used paracetamol with an average dose of 1044 mg, while 21 patients on buprenorphine used an average dose of 1057 mg paracetamol per day.

When we analyzed equianalgesic TD buprenorphine dose as 1:100, there was a significant difference in starting and final opioid doses among the two groups, which was unexpected considering that both groups have virtually the same patients characteristics. When we calculated the buprenorphine equianalgesic ratio to 1:80, the OMEs were similar between the two groups.

Finally, there was no difference in time spent in hospice between the groups using fentanyl or buprenorphine TD patch (16.40 versus 14.98 days). Although a trend was present between the TD group and the group with only peroral opioids, the difference in survival was not statistically significant.

We additionally analyzed the differences when TD opioids were combined with peroral opioids and NSAIDs (Table 2). Unsurprisingly, if the patients had additional peroral opioids in therapy, they had a higher starting and final OME dose per day. On the other hand, the addition of NSAIDs did not alter either starting of final opioid dose. However, no combination of adjuvant peroral opioids or NSAIDs affected the length of stay in the hospice. There was a trend of longer stay in the fentanyl + NSAIDs group. However, this could be because this group had the best performance score, which directly correlates to survival.

## 4. Discussion

To the best of our knowledge, our data represent the first direct comparison of different TD opioids in a hospice environment.

We initially calculated and compared buprenorphine to fentanyl using a 1:100 conversion rate, since there are no definite recommended conversion rates [13]. The ratio of 1:100 we initially used for buprenorphine produced statistically significant changes in opioid doses between the fentanyl and buprenorphine group of patients. However, it is obvious from Table 1 that there were virtually no differences in patient characteristics between the two patient groups. Hence, it is likely that the patients were using similar OME doses. We modified the analysis to use the 1:80 conversion rate for buprenorphine, and judging by our results this is probably the more appropriate conversion rate for elderly hospice patients.

However, regarding safety, it was important to note that there were no differences in survival between the two groups, and both seem safe for hospice use in elderly. A trend towards a longer survival with TD opioids, compared to peroral ones, was observed but was not statistically significant.

The safety did not differ even with the addition of NSAIDs or peroral opioids to TD opioids. Surprisingly, however, the patients who used NSAIDs concomitantly did not have a lower initial or final dose. This might support the thesis that the only essential analgesics in the hospice are the opioids (2).

Several weaknesses of the study need to be mentioned. First of all, we did not analyze the quality of life or pain ratings in this study, which is paramount for the choice of opioid and the dose elevation. However, our goal here was only to access the relationship of different TD opioids and their combinations to survival, regardless of the effect on the quality of life or symptoms such as constipation or delirium. We also recognize that there may be unknown confounders between the groups that receive opioids versus opioids plus NSAIDs which may account for the lack of opioid sparing effects. Additionally, this was a retrospective investigation, enrolling only Caucasian patients and performed in only one center. For future studies, to eliminate potential bias, we recommend performing a multicentric randomized prospective investigation with a parallel analysis of the quality of life and the doses and formulation of opioids.

## 5. Conclusion

This research supports the safety of both TD fentanyl and buprenorphine in the hospice population. The choice of the drug should depend on patient preferences, dosing schedule, and the type of pain. Although adjuvant NSAIDs could be useful in the treatment of pain in terminal cancer, they do not affect survival or the reduce the opioid doses. Finally, our data suggest that for elderly hospice patients, with cancer as a dominant diagnosis, the 1:80-1:85 equianalgesic ratio of buprenorphine might be more appropriate for conversion purposes.

## Figures and Tables

**Table 1:** Differences between patients on transdermal fentanyl and buprenorphine.

Parameter	Fentanyl (1:100)	Buprenorphine (1:100)	Buprenorphine (1:80)	Peroral opioids
Number of patients	292	93		88
Average age (yr)	76.27 ± 6.75	76.68 ± 6.59		78.64 ± 7.42
Female (%)	150 (51.4)	45 (48.4)		41 (46.6)
Cancer patients (%)	272 (93.1)	88 (94.6)		73 (82.9)
Number of patients died (%)	261 (89.4)	83 (89.2)		74 (84.1)
Initial opioid dose (OME)	105.21 ± 91.80	123.29 ± 104.46	102.11 ± 89.16	15.07 ± 17.47
Last opioid dose (OME)	144.30 ± 97.30	161.17 ± 104.02	133.76 ± 89.97	18.15 ± 18.63
Absolute difference	39.09	37.88	31.65	3.08
Percentage difference	37.1	23.5	30.99	20.4
Patients with dose elevation (%)	120 (41.1)	38 (40.8)		15 (17.0)
Patients with dose reduction (%)	3 (1.0)	1 (1.1)		3 (3.4)
Days in hospice ± SD	16.40 ± 19.36	14.98 ± 17.82		12.86 ± 15.78
Performance score ± SD	3.19 ± 0.96	3.25 ± 0.85		3.22 ± 0.92
Additional peroral opioids (%)	222 (76.0)	68 (73.1)		n/a
Additional anxiolytic (%)	154 (52.7)	44 (47.3)		36 (40.9)
Additional NSAID (%)	108 (36.9)	33 (35.5)		47 (53.4)
Additional antipsychotic (%)	91 (31.2)	30 (32.2)		33 (37.5)

OME = oral morphine equivalent, mg/day. NSAID = nonsteroidal anti-inflammatory drugs.

The performance score is rated from 0 to 4, with 0 being fully mobile patients and 4 being bedridden patients.

**Table 2:** Differences in dosages and survival in patients with transdermal opioids with additional peroral analgesics (with an equianalgesic ratio of 1:80 for buprenorphine).

Combination	Number of patients	Initial dose	Final dose	Diff. (%)	Survival (SD)	Performance score
Fentanyl + PO opioids	222	110.54 ± 97.66	154.77 ± 103.48	44.23 (40.0)	16.55 ± 19.28	3.15 ± 0.96
Buprenorphine + PO opioids	68	117.35 ± 95.49	151.74 ± 96.49	34.39 (29.3)	16.48 ± 19.14	3.26 ± 0.80
P value	-	0.37	0.92	0.81	0.89	0.60
Fentanyl without PO opioids	70	88.28 ± 67.93	111.09 ± 64.50	22.80 (25.8)	15.91 ± 19.75	3.36 ± 0.92
Buprenorphine without PO opioids	25	60.67 ± 50.70	84.86 ± 40.39	24.19 (39.9)	10.88 ± 13.07	3.20 ± 1.00
P value	-	0.68	0.74	0.57	0.22	0.48
Fentanyl + NSAID	108	107.36 ± 99.35	160.13 ± 104.27	52.77 (49.1)	19.76 ± 21.29	3.05 ± 0.95
Buprenorphine + NSAID	33	102.78 ± 78.09	140.33 ± 79.26	37.56 (36.5)	14.96 ± 13.20	3.24 ± 0.79
P value	-	0.81	0.48	0.65	0.21	0.39
Fentanyl without NSAID	184	103.95 ± 87.33	135.01 ± 91.99	31.07 (29.89)	14.43 ± 17.89	3.28 ± 0.96
Buprenorphine without NSAID	60	101.75 ± 95.34	130.15 ± 95.80	28.39 (27.90)	14.98 ± 20.02	3.25 ± 0.89
P-value	-	0.85	0.89	0.65	0.80	0.59

Doses are presented oral morphine equivalents, mg/day. NSAID = nonsteroidal anti-inflammatory drugs. PO = per oral. The performance score is rated from 0 to 4, with 0 being fully mobile patients and 4 being bedridden patients.

## Data Availability

The data is in control of the authors and is available after contacting the corresponding author.
